# Glutamine Deprivation Synergizes the Anticancer Effects of Cold Atmospheric Plasma on Esophageal Cancer Cells

**DOI:** 10.3390/molecules28031461

**Published:** 2023-02-02

**Authors:** Wei Zhao, Xumiao Jing, Tao Wang, Fengqiu Zhang

**Affiliations:** 1 Henan Key Laboratory of Ion-Beam Bioengineering, School of Physics and Microelectronics, Zhengzhou University, Zhengzhou 450001, China; 2 College of Nursing and Health, Zhengzhou University, Zhengzhou 450001, China; 3 Telethon Kids Institute, Perth, WA 6872, Australia; 4 School of Medicine, University of Western Australia, Perth, WA 6872, Australia

**Keywords:** esophageal cancer, cold atmospheric plasma (CAP), reactive oxygen species (ROS), glutamine deprivation, synergistic treatment

## Abstract

Esophageal cancer is a highly aggressive malignancy with a low response to standard anti-cancer therapies. There is an unmet need to develop new therapeutic strategies to improve the clinical outcomes of current treatments. Cold atmospheric plasma (CAP) is a promising approach for cancer treatment, and has displayed anticancer efficacy in multiple preclinical models. Recent studies have shown that the efficacy of CAP is positively correlated with intracellular reactive oxygen species (ROS) levels. This suggests that aggressively increasing intracellular ROS levels has the potential to further improve CAP-mediated anticancer efficacy. Glutamine plays an important role in cellular ROS scavenging after being converted to glutathione (GSH, a well-described antioxidant) under physiological conditions, so reducing intracellular glutamine levels seems to be a promising strategy. To test this hypothesis, we treated esophageal cancer cells with CAP while controlling the supply of glutamine. The results showed that glutamine did affect the anticancer effect of CAP, and the combination of CAP stimulation and glutamine deprivation significantly inhibited the proliferation of esophageal cancer cells compared to the control group (*p* < 0.05). Furthermore, flow cytometric analysis documented a significant increase in more than 10% in apoptosis and necrosis of esophageal cancer cells after this synergistic treatment compared to the control group (*p* < 0.05). Thus, these results provide the first direct evidence that the biological function of CAP can be modulated by glutamine levels and that combined CAP stimulation and glutamine deprivation represent a promising strategy for the future treatment of esophageal cancer.

## 1. Introduction

Esophageal cancer is among the leading causes of cancer-related deaths worldwide [1,2,3]. The current mainstream treatments for esophageal cancer include esophagectomy, chemotherapy, radiotherapy, targeted therapy, multimodal therapy and recently, immunotherapy [4]. However, the prognosis of esophageal cancer remains unsatisfactory [5], novel therapeutic approaches are highly demanded to improve the treatment outcomes of this group of patients.

Plasma is the fourth state of matter in addition to solid, liquid and gas, and is usually in the form of partially ionized gas (consisting of ions, electrons and neutral particles) [6,7]. In recent years, plasma generated at near room temperature, termed cold atmospheric plasma (CAP), has been introduced [8]. There are two main types of CAP devices, namely plasma jet and dielectric barrier discharge (DBD), and the DBD device is more suitable for bulk preparation; therefore, it is more suitable for clinical application [7,9]. Over the past decade, CAP has shown potential in the treatment of different types of cancers [10,11,12,13,14]. The current understanding of the mechanisms behind CAP-related anti-cancer effects suggests that CAP includes not only electric fields, ions and electrons, but also cytotoxic reactive oxygen species (ROS) to specifically damage intracellular structures of cancer cells [15,16]. In addition, it was also found that CAP mediated cancer inhibition involves the stimulation of intracellular reactive oxygen and nitrogen species (RONS), and the pretreatment with ROS scavenger N-acetyl-L-cysteine (NAC) revealed the significant impact of ROS in cancer treatment [17]. Importantly, such CAP-produced ROS displayed desirable anti-cancer potential as they demonstrated only negligible toxic effects on normal tissues, implicating a targeted effect of CAP treatment to cancer [18]. This observed cancer-specificity is understandable, compared to normal cells, cancer cells have a higher metabolism rate (therefore a higher basal ROS level), which makes them naturally susceptible to exogenous ROS-caused apoptosis than normal cells [8]. Furthermore, cancer cells contain more water channel proteins and less cholesterol in their cell membranes (higher membrane permeation), which facilitates the diffusion of CAP-generated ROS through the membrane and exert their biological functions [11,19,20,21].

Apart from the direct cytotoxicity of ROS, CAP-mediated anticancer effects also benefit from ROS-mediated intracellular scavenging. As previously reported, ROS could remove critical antioxidant components (e.g., superoxide dismutase, glutathione peroxidase, glutathione) of cells and induce severe cellular oxidative damage [22]. Among these antioxidants, the removal of glutathione, a built-in cell protecting agent (being able to eliminate cytotoxic O_2_^−^ radicals), is of particular importance for cell death [23]. Based on this knowledge, we asked if reducing cellular glutathione level, thereby removing glutathione-based cell protection, could synergize CAP treatment. This hypothesis is further supported by the finding that compared with normal cells, glutathione levels showed a significant increase in different types of tumor cells (suggesting glutathione depletion could specifically affect tumor cells) [24]. However, directly reducing glutathione is a very hard task. As an alternative way, depleting glutamine looks more manurable. This is primarily because glutamine is a precursor for glutathione synthesis, depletion of glutamine would theoretically lead to glutathione exhaustion. In addition, glutamine-targeted treatment is clinically feasible, due to the fact that glutamine is not only one of the most common supplements for cell culture (easily be tested in in vitro assay), but also there are various types of glutamine inhibitors available for future in vivo and clinical trials.

In the current study, we aim to further improve the efficacy of CAP-based esophageal cancer treatment, through a combined CAP and glutamine exhaustion treatment. Indeed, our findings suggest that CAP and glutamine starvation can effectively treat esophageal cancer in vitro models. Importantly, given the similar effects of ROS and glutamine inhibition on different cell types, the results collected in this study could be translated to the treatment of other cancers as well.

## 2. Results

### 2.1. CAP Reduced the Viability of Esophageal Cancer

**Figure 1:**
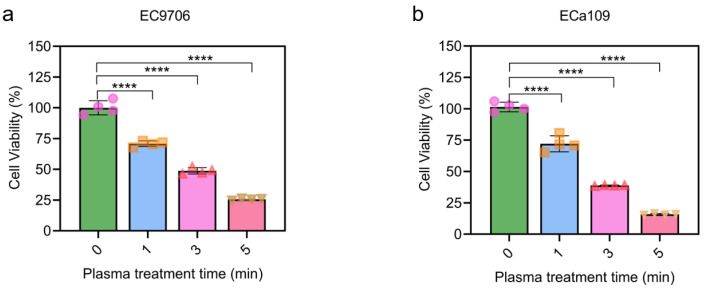
Cell viability decreased gradually after CAP treatment for 0, 1, 3, 5 min. (**a**). Cell viability of EC9706 cells; (**b**). Cell viability of ECa109 cells. **** *p* < 0.0001. The different symbols (circle, square, triangle and inverted triangle) represent different experiment repeats of the same treatment.

In this work, the viability of esophageal cancer after CAP treatment for different timepoints were examined using the MTT assay. As shown in Figure 1, CAP could significantly inhibit cell proliferation in a time-dependent manner in both EC9706 and ECa109 cells. For example, after 1, 3, 5 min CAP treatment (followed by a 24 h incubation), EC9706 cells displayed a notable decrease in viability compared to the untreated cells (0 min), with the 5 min treatment showing a 4-fold decrease (Figure 1a). A similar phenomenon was also recorded with ECa109 cells (Figure 1b). As a result, the treatment time of CAP was selected as 5 min in the subsequent studies.

### 2.2. CAP Notably Increased RONS Level in Cell Culture Medium

**Figure 2:**
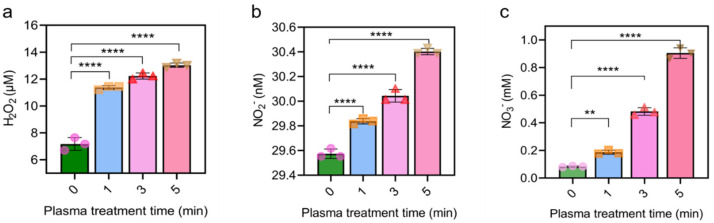
RONS assay after CAP treatment of different timepoints. (**a**). Content of H_2_O_2_ in the medium after 0, 1, 3, 5 min treatment; (**b**). Content of NO_2_^−^ in the medium after 0, 1, 3, 5 min treatment; (**c**). Content of NO_3_^−^ in the medium after 0, 1, 3, 5 min treatment. ** *p* < 0.01 and **** *p* < 0.0001. The different symbols (circle, square, triangle and inverted triangle) represent different experiment repeats of the same treatment.

Due to the rapid quenching of short-lived reactive species (very short half-life), the long-lived hydrogen peroxide (H_2_O_2_), nitrite (NO_2_^−^) and nitrate (NO_3_^−^) were considered to be the three main RONS for the anti-tumor effects of CAP [25]. Therefore, the levels of these three species in the cell culture medium were quantified. As shown in Figure 2, after CAP treatment (0, 1, 3, 5 min), the levels of H_2_O_2_, NO_2_^−^ and NO_3_^−^ in the plasma-activated mediator displayed a time-dependent increase. The increase in the tested RONS was especially obvious after 5 min plasma treatment. For example, compared with the untreated group, H_2_O_2_ increased from 7.17 to 13.03 μM, NO_2_^−^ increased from 29.57 to 30.39 nM, and NO_3_^−^ increased more than 10 folds from 0.08 to 0.91 mM.

### 2.3. CAP-Induced Long-Lasting Reactive Species Inhibited the Viability of Esophageal Cancer

**Figure 3:**
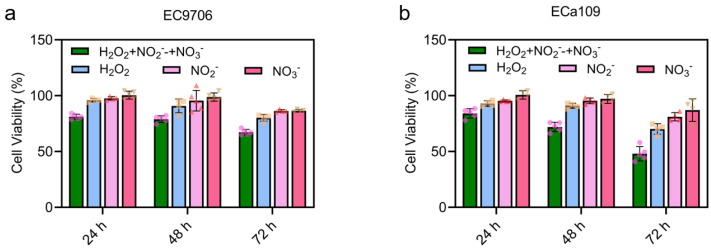
Cell viability after CAP-stimulated RONS treatment. (**a**). Cell viability of EC9706 cells after 24 h, 48 h, 72 h treatment with H_2_O_2_, NO_2_^−^ and NO_3_^−^; (**b**). Cell viability of ECa109 cells after 24 h, 48 h, 72 h treatment with H_2_O_2_, NO_2_^−^ and NO_3_^−^. The concentration of H_2_O_2_, NO_2_^−^ and NO_3_^−^ were 13.03 µM, 30.39 nM and 0.91 mM, respectively. The different symbols (circle, square, triangle and inverted triangle) represent different experiment repeats of the same treatment.

To investigate the inhibition effects of the three major CAP-induced long-lasting reactive species (H_2_O_2_, NO_2_^−^ and NO_3_^−^) on esophageal cancer, ECa109 and EC9706 cells were cultured with medium containing 13.03 μM H_2_O_2_, 30.39 nM NO_2_^−^ and 0.91 mM NO_3_^−^ (equivalent to the concentrations after 5 min CAP stimulation) for 24 h, 48 h and 72 h, respectively. As shown in Figure 3, after 24 h and 48 h of incubation, the cell viability of ECa109 and EC9706 decreased only slightly. However, after 72 h incubation, both cell lines showed a significant decrease in viability. In addition, it was also found that H_2_O_2_ may inhibit cells more effectively than NO_2_^−^ and NO_3_^−^, though a significant difference was not recorded (Figure 3).

Apart from individual treatment, we also conducted a combined treatment with 13.03 μM H_2_O_2_, 30.39 nM NO_2_^−^ and 0.91 mM NO_3_^−^ for 24 h, 48 h and 72 h, as shown in Figure 3. Interestingly, it seemed that the combined treatment achieved a synergistic effect compared with the individual groups, especially after 72 h of treatment. As shown, in EC9706 cells, 67.35% viability was observed after combined treatment, in contrast to the viability rate of 80.16%, 86.37% and 86.45% for H_2_O_2_, NO_2_^−^ and NO_3_^−^ respectively. Similarly, in ECa109 cells, 48.01% viability was recorded for combined treatment and 70.21%, 81.11%, 87.10% for H_2_O_2_, NO_2_^−^ and NO_3_^−,^ respectively. This suggests that an unknown mechanism exists for RONS mediated cytotoxicity. At the same time, this also suggests that it may be important to monitor the concentration of all these RONs after CAP treatment.

### 2.4. Glutathione Reduced the H_2_O_2_ Level in the CAP Activated Medium

**Figure 4:**
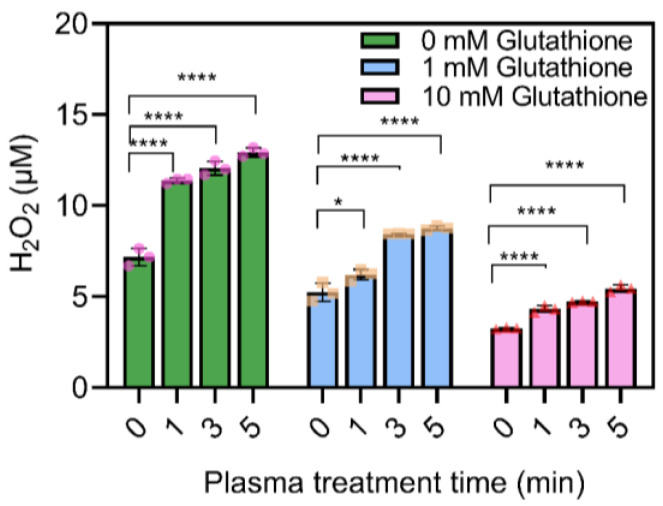
H_2_O_2_ assay in CAP activated medium after glutathione (GSH) treatment. * *p* < 0.05 and **** *p* < 0.0001. The different symbols (circle, square and triangle) represent different experiment repeats of the same treatment.

H_2_O_2_ often imposes oxidative stress on cells and tissues. As it has been reported that H_2_O_2_ could be depleted by glutathione in lung cancer cells [26], we tested this effect in our esophageal cell models by adding it into cell culture medium after CAP treatment. As shown in Figure 4, the medium supplemented with glutathione showed a clear dose-dependent H_2_O_2_ decrease. Specifically, compared to the untreated control group, the addition of both 1 mM and 10 mM of glutathione showed a significant H_2_O_2_ decrease, and the 10 mM group observed a nearly 2-fold decrease in H_2_O_2_ amount than the 1 mM group (comparison was made by comparing the same timepoint of CAP treatment). This result indicates that glutathione can not only deplete the H_2_O_2_ produced in cells as previously reported, but also exhaust H_2_O_2_ created by CAP stimulation.

### 2.5. Cell Viability Decreased after Combined CAP Stimulation and Glutamine Deprivation

Glutamine is a precursor of glutathione in cells [27]. Since glutathione can reduce H_2_O_2_, an important factor for CAP mediated cytotoxicity, we suspect that glutamine may also affect CAP treatment. To test this hypothesis, a combinatorial treatment of CAP stimulation and glutamine deprivation was performed.

**Figure 5:**
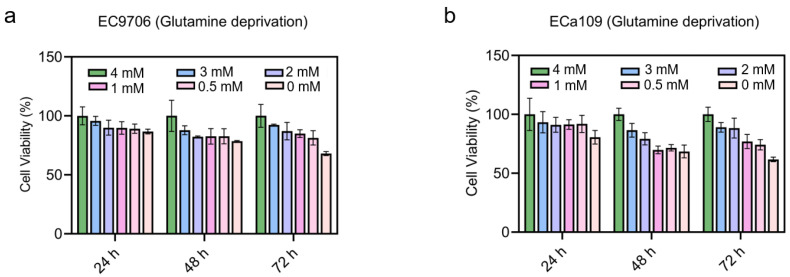
Cell viability after glutamine deprivation. (**a**). Cell viability of EC9706 cells after glutamine deprivation for 24 h, 48 h, and 72 h. (**b**). Cell viability of ECa109 cells after glutamine deprivation for 24 h, 48 h, and 72 h. The normal glutamine concentration for cell growth is 4 mM.

Firstly, a glutamine deprivation assay was carried out to analyze the effect of different glutamine concentrations on the proliferation of esophageal cancer cell line. After 24 h glutamine deprivation, as demonstrated in Figure 5a, compared with normal glutamine treatment (i.e., 4 mM), EC9706 cells showed no significant difference in cell viability after 2 mM and 3 mM glutamine treatment, while the 0.5 mM and 1 mM glutamine treated cells showed a significant decrease in cell viability. After 48 h and 72 h deprivation, the viability of both cell lines gradually decreased in a glutamine dose-dependent manner (Figure 5). The strongest inhibition was found in cells treated with 0 mM glutamine compared to the control (normal conditions culture with 4 mM glutamine). These results provided direct evidence that glutamine deprivation could inhibit the proliferation of esophageal cancer.

**Figure 6:**
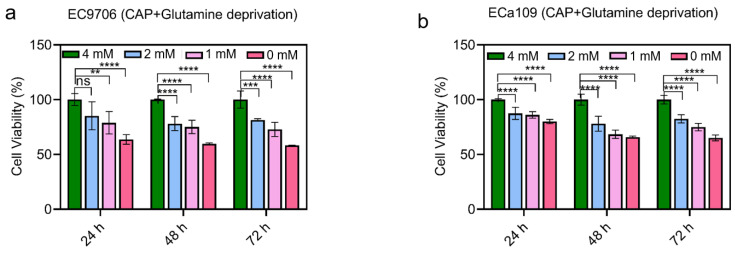
Cell viability after 5 min CAP treatment combined with glutamine deprivation. (**a**). Cell viability of EC9706 cells after CAP treatment combined with glutamine deprivation; (**b**). Cell viability of ECa109 cells after CAP treatment combined with glutamine deprivation. 4 mM glutamine is the normal concentration in the culture medium. “ns” means *p* > 0.05, ** *p* < 0.01, *** *p* < 0.001 and **** *p* < 0.0001.

Based on these results, we next combined CAP stimulation and glutamine deprivation to treat esophageal cancer and analyzed the cell viability. Figure 6 shows that compared to CAP treatment alone (cells treated with 5 min CAP but live in 4 mM glutamine), the combinatorial treatment of CAP stimulation and glutamine deprivation significantly reduced the viability of both cell lines in a dose-dependent manner. This work indicated that the protection of glutamine (e.g., 4 mM glutamine) to cells involves the removal of reactive species, and CAP treatment combined with glutamine deprivation might synergize the tumor inhibition effect.

### 2.6. Combined CAP Stimulation and Glutamine Deprivation Reduced Glutathione Level

**Figure 7:**
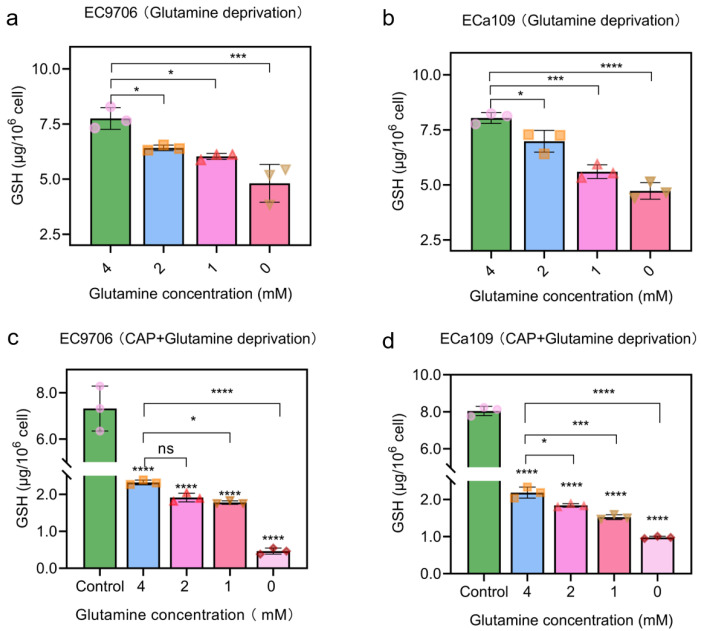
Glutathione (GSH) quantification after 5 min CAP treatment combined with glutamine deprivation. (**a**). Glutathione level of EC9706 cells after glutamine deprivation; (**b**). Glutathione level of ECa109 cells after glutamine deprivation; (**c**). Glutathione level of EC9706 cells after 5 min CAP treatment with glutamine deprivation; (**d**). Glutathione level of ECa109 cells after 5 min CAP treatment with glutamine deprivation. Control represents that glutamine is 4 mM without CAP treatment. “ns” means *p* > 0.05, “*” means * *p* < 0.05, “***” means *** *p* < 0.001 and “****” means **** *p* < 0.0001. The different symbols (circle, square, triangle, inverted triangle and diamond) represent different experiment repeats of the same treatment.

Glutathione has the physiological function of scavenging free radicals, antioxidant and eliminating electrophile [28]. In order to analyze the antioxidant influence of glutamine deprivation on CAP treatment, firstly we analyzed the change in cellular glutathione amount. Figure 7a,b show that the intracellular glutathione level decreased gradually after glutamine deprivation compared to that of the control cells (treated with 4 mM glutamine). Figure 7c and d show that glutathione level was significantly lower after combined CAP stimulation and glutamine deprivation, compared to that of the CAP stimulation alone (cells lived in 4 mM glutamine). These results indicate that glutamine deprivation reduced the feedstock source of glutathione, and CAP treatment may inhibit the production of glutathione or delete a certain amount of glutathione. The implication of all these is that the combined CAP stimulation and glutamine deprivation may lead to enhanced inhibition of cancer cell line partly because of glutathione exhaustion.

### 2.7. Combined CAP Stimulation and Glutamine Deprivation Improved ROS Levels

Currently, it is believed that CAP inhibits cancer cell proliferation partly through increasing intracellular RONS (reactive oxygen and nitrogen species), which are mainly maintained by ROS (reactive oxygen species), and the high level of ROS can cause cell damage or even death [17]. Glutathione is one of the main intracellular scavengers of ROS [28]; therefore, the following work was carried out to analyze intracellular ROS change after CAP treatment and glutamine deprivation.

**Figure 8:**
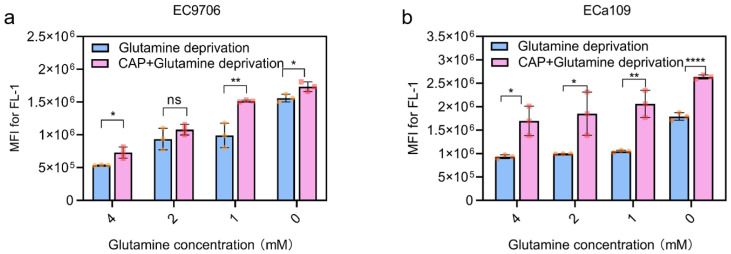
Intracellular ROS quantification after glutamine deprivation or CAP treatment combined with glutamine deprivation. (**a**). ROS level of EC9706 cells; (**b**). ROS level of ECa109 cells. MFI stands for mean fluorescence intensity of ROS. “ns” means *p* > 0.05, “*” means * *p* < 0.05, “**” means ** *p* < 0.01, and “****” means **** *p* < 0.0001. The different symbols (circle and square) represent different experiment repeats of the same treatment.

Figure 8 shows a dose-dependent relationship between intracellular ROS level and glutamine deprivation: the lower the glutamine level was, the higher the ROS level was observed. In addition, the ROS level of CAP combined with glutamine deprivation was significantly higher than that of the CAP treatment alone (4 mM glutamine) and glutamine deprivation alone groups, indicating a synergistic effect of CAP stimulation and glutamine deprivation in the up-regulation of ROS, which may be responsible for cell inhibition.

### 2.8. Intracellular H_2_O_2_ Increased after Combined CAP Stimulation and Glutamine Deprivation

**Figure 9:**
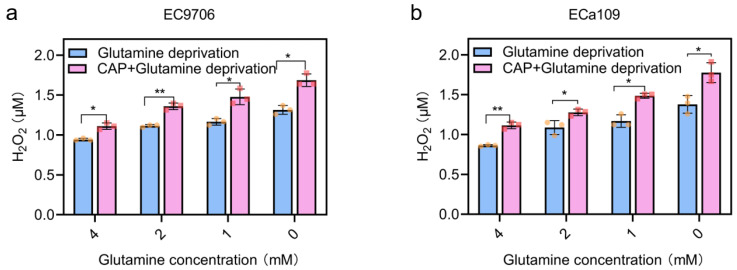
Intracellular H_2_O_2_ quantification after glutamine deprivation or CAP treatment combined with glutamine deprivation. (**a**). H_2_O_2_ level of EC9706 cells; (**b**). H_2_O_2_ level of ECa109 cells. “*” means * *p* < 0.05 and “**” means ** *p* < 0.01. The different symbols (circle and inverted triangle) represent different experiment repeats of the same treatment.

ROS generated during CAP treatment, such as H_2_O_2_, have been found to mediate tumor-specific mitochondrial network collapse and cytotoxicity in a tumor-specific manner [29]. Figure 3 indicates that H_2_O_2_ was the most toxic ROS among the three major long-lasting reactive species produced by CAP. Glutathione can provide electrons to enzymes such as glutathione peroxidase to reduce H_2_O_2_ into H_2_O [23]. Therefore, intracellular H_2_O_2_ levels were measured in esophageal cancer after glutamine deprivation alone or CAP treatment combined with glutamine deprivation. Figure 9 shows that there was a dose-dependent relationship between intracellular H_2_O_2_ level and glutamine deprivation, with increased H_2_O_2_ level in the 0 mM glutamine group compared to the 4 mM glutamine group in both tested esophageal cancer, with an increase from 0.94 μM to 1.31 μM for EC9706 cells and from 0.86 μM to 1.38 μM for ECa109 cells. In addition, the intracellular H_2_O_2_ level was significantly higher after combined CAP stimulation and glutamine deprivation than that of the CAP treatment alone (4 mM glutamine) or glutamine deprivation alone group. Specifically, compared to 0 mM glutamine alone treated EC9706 cells, as shown in Figure 9, a significant increase (from 1.31 μM to 1.69 μM) in H_2_O_2_ level was recorded in the CAP combined with 0 mM glutamine group, and for ECa109 cells, the CAP combined with 0 mM glutamine showed a significant increase in H_2_O_2_ from 1.38 μM to 1.78 μM compared to 0 mM glutamine alone. This result indicated that the combination of CAP stimulation and glutamine deprivation had a synergistic effect on H_2_O_2_ production.

### 2.9. MAD Increased after CAP Treatment Combined with Glutamine Deprivation

**Figure 10:**
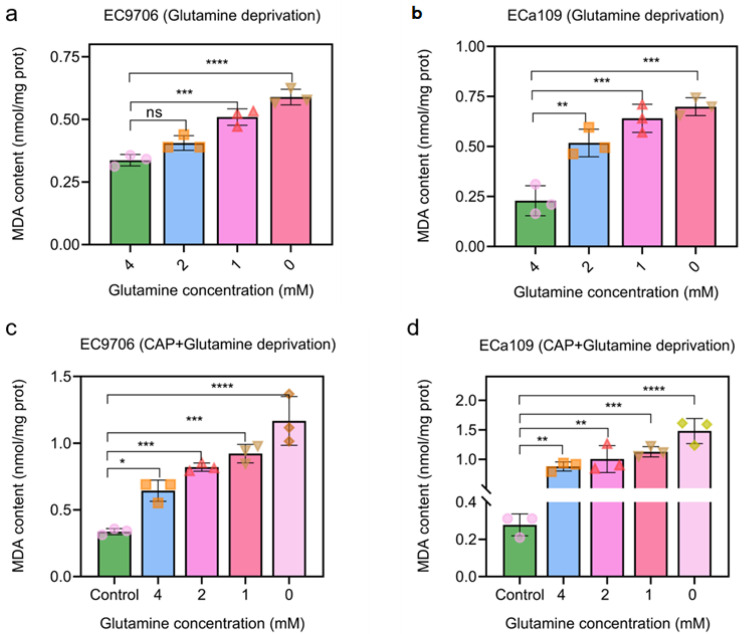
MDA levels of esophageal cancer. (**a**,**b**). MDA levels of EC9706 cells and ECa109 cells after glutamine deprivation; (**c**,**d**). MDA levels of EC9706 cells and ECa109 cells after CAP treatment combined with glutamine deprivation. Control represents that glutamine is 4 mM without CAP treatment. “ns” means *p* > 0.05, “*” means * *p* < 0.05, “**” means ** *p* < 0.01, “***” means *** *p* < 0.001 and “****” means **** *p* < 0.0001. The different symbols (circle, square, triangle, inverted triangle and diamond) represent different experiment repeats of the same treatment.

It is well known that ROS could cause oxidative damage to cells. The reactive species may alter the structure of the cell membrane and lead to lipid peroxidation of polyunsaturated fatty acids. Malondialdehyde (MDA) is one of the most common and harmful products of lipid peroxidation and may lead to cellular damage [30]. Therefore, the MDA assay can be used to estimate the intensity of oxidative stress and lipid peroxidation. Figure 10a,b shows that MDA levels were significantly increased in both EC9706 and ECa109 cells of the 0 mM glutamine treated group compared to the 4 mM glutamine treated group, with the MDA level increasing from 0.34 nmol/mg prot to 0.59 nmol/mg prot in EC9706 cells and from 0.23 nmol/mg prot to 0.70 nmol/mg prot in ECa109 cells. The results in Figure 10c,d show that MDA levels in esophageal cancer were significantly increased after combined CAP and glutamine deprivation treatment compared to glutamine deprivation alone or CAP treatment alone (cells lived in 4 mM glutamine), as shown, combined CAP and glutamine deprivation (0 mM glutamine) treatment increased the MDA level of EC9706 cells to 1.17 nmol/mg prot and combined CAP and glutamine deprivation (0 mM glutamine) treatment increased the MDA level of ECa109 cells to 1.48 nmol/mg prot. These results suggested that combined treatment of CAP and glutamine deprivation could increase the level of oxidative damage in esophageal cancer.

### 2.10. Apoptosis and Necrosis Increased after Combined Treatment of CAP Stimulation and Glutamine Deprivation

**Figure 11:**
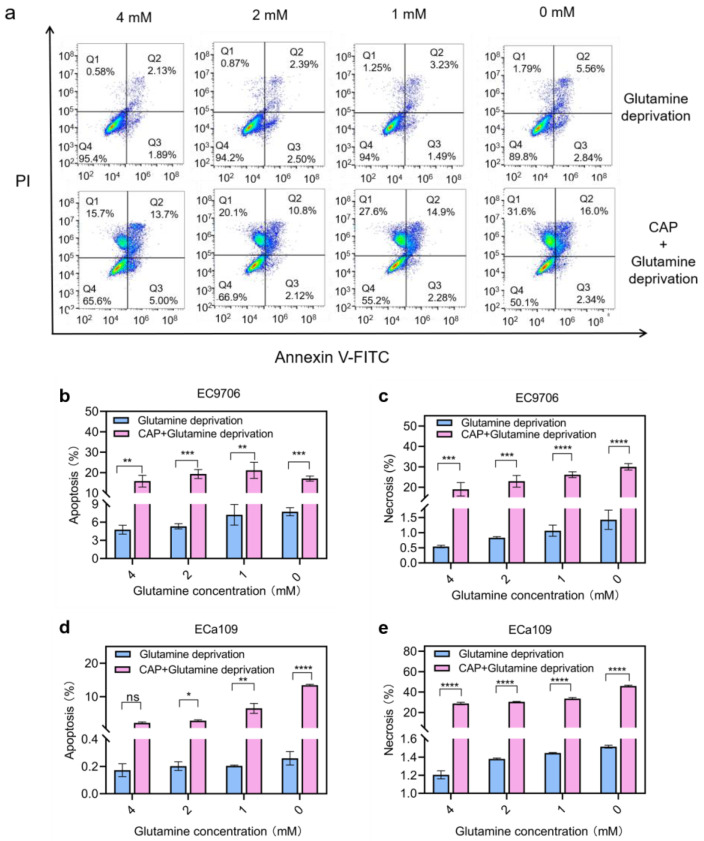
Apoptosis and necrosis assay for esophageal cancer after CAP treatment combined with glutamine deprivation. (**a**). Flow cytogram of ECa109 cells after glutamine deprivation alone or CAP treatment combined with glutamine deprivation; (**b**,**c**). Apoptosis and necrosis assay for EC9706 cells after glutamine deprivation alone or CAP treatment combined with glutamine deprivation; (**d**,**e**). Apoptosis and necrosis assay for ECa109 cells after glutamine deprivation alone or CAP treatment combined with glutamine deprivation. “ns” means *p* > 0.05, “*” means * *p* < 0.05, “**” means ** *p* < 0.01, “***” means *** *p* < 0.001 and “****” means **** *p* < 0.0001.

Based on our above observation that there was an increase in ROS, H_2_O_2_ and MDA in cancer cells after CAP treatment combined with glutamine deprivation (which would lead to apoptosis and even necrosis of the tumor cells [31]), further apoptosis and necrosis analysis were carried out. Figure 11b,d shows that the CAP combined with glutamine deprivation group of EC9706 and ECa109 cells showed a significant increase in apoptosis compared to the glutamine deprivation alone group, with the combined treatment showing an increase in apoptosis from 7.74% to 17.11% in EC9706 cells, compared to the 0 mM glutamine treatment group alone. Similarly, in ECa109 cells, the combined treatment increased apoptosis from 0.26% to 13.45% compared to glutamine depletion alone. Figure 11c,e shows that for EC9706 cells, the combined treatment resulted in a significant increase in cell necrosis from 1.43% to 30% compared to glutamine deprivation alone, and for ECa109 cells, the corresponding data increased notably from 1.52% to 45.90%. These results suggest that glutamine deprivation and CAP have a synergistic effect on apoptosis and necrosis in esophageal cancer.

## 3. Discussion

CAP has shown great anti-cancer potential in recent years. As reported, CAP could selectively kill cancer cells, and significantly inhibit the growth and metastasis of tumors in multiple models [32]. The current understanding of CAP-mediated anticancer effect suggests that CAP can produce cytotoxic RONS and cause cell death through oxidative damage [33]. Cellular damage induced by oxidative stress is currently of great interest. For example, Ji et al. provided a promising method for tumor microenvironment regulation by using ferrocene-containing nucleic acid-based energy storage nanoagents to convert the light energy stored in the reagents [34]. In another example, Chen et al. used MB-encapsulated ZIF-90 (ZIF-90/MB) to selectively release MB in mitochondria under light by generating singlet oxygen (^1^O_2_) [35]. Indeed, RONS originated from CAP include multiple reactive species such as nitric oxide (NO), superoxide (O_2_^−^), hydrogen peroxide (H_2_O_2_), singlet oxygen (^1^O_2_), ozone (O_3_), and even hydroxyl radicals (OH•). These reactive species can cause peroxidation of cellular double lipid membranes and lead to apoptosis [36,37]. Among these species, three of the long-lived reactive species (i.e., H_2_O_2_, NO_2_^−^ and NO_3_^−^) play the most important roles in CAP-based anti-cancer treatment [15,25,38,39]. For example, Dehui Xu et al. demonstrated that H_2_O_2_ and O_2_^−^ were the main reactants of plasma treatment [40]. In another case, it was also found that H_2_O_2_ in ROS had the most significant inhibitory effect on different types of cancer cells [20].

However, using CAP alone only resulted in limited treatment outcomes. This is because of the ubiquitous existence of glutathione in cells, which could down-regulate CAP-produced RONS, the main killing mechanism of CAP treatment [41,42,43].

To develop CAP as a clinically available therapy, different combinatorial strategies have been practiced and shown promising results in different studies. For example, in a recent study, it was found that CAP could be used as an adjuvant to immunotherapy in the treatment of glioblastoma multiforme through ROS-induced upregulation of the immune system [44]. At the same time, combined CAP and chemotherapy have also been tested. For instance, J, K et al. found that the combination of CAP and temozolomide treatment could successfully reverse the drug resistance of glioma cells to temozolomide and promote cell cycle arrest compared to temozolomide alone [45,46]. As reported by R, M and colleagues, the benefit of combined CAP and temozolomide might due to the regulation of antioxidant-specific glutathione (GSH)/glutathione peroxidase 4 (GPX4) signaling [47,48]. Similar results were obtained when CAP was used together with cisplatin in three different cell lines (i.e., Cal27, FaDu and OSC19 cells) [49].

Compare with some existing combined CAP treatments (such as chemo combination, radio-combination), combined CAP and glutathione deprivation represents obvious advantages due to the endogenous synergistic anticancer effects. One of the significances of this work lie in the fact that it provides a clinically practicable method for combined CAP and glutathione depletion treatment. The present study differs from previous combined CAP and glutathione depletion treatments such as the one developed by using gold nanoparticles (which reduces intracellular glutathione and thus makes cells more susceptible to ROS production [8]) for enhanced CAP-induced apoptosis, this work achieved an indirect glutathione depletion by targeting glutamine, the precursor of glutathione. In fact, our experimental design is similar to this work. However, rather than directly targeting glutathione, we achieved an indirect glutathione depletion by targeting glutamine, the precursor of glutathione. This is important as compared with the hard-to-target glutathione (in a clinical setting), there are various types of commercial glutamine inhibitors (e.g., glutamine transporter inhibitor V-9302) available for the clinical translation of the combined CAP and glutamine depletion treatment (through targeted therapy to delivery such inhibitors to directly to tumors to avoid glutamine-depletion associated side-effects to normal cells).

Glutamine is the main carbon source for the biosynthesis of nucleotides and non-essential amino acids and is extremely important for cell growth and proliferation. In addition, as a precursor of glutathione, it provides NAD(P)H to cells to help neutralize intracellular ROS [50,51,52] and maintain redox homeostasis [53,54]. Importantly, it has been reported that compared with normal cells, the survival of cancer cells depends more on glutamine supply. This is because of the improved metabolism (to support the fast growth) of cancer cells, which needs considerable glutamine/glutathione than normal cells. As shown, glutamine is highly active in many human cancers, including triple-negative breast cancer, vulvar cancer, lymphoma, pancreatic cancer and lung cancer [55,56]. These demonstrate the rationale of glutamine-targeted treatment. The rationale of combined CAP and glutamine depletion treatment is also supported by the observation that glutamine itself has an anti-apoptotic function through increasing the expression of Hsp70, an anti-apoptotic agent [57].

According to our experiments, glutamine deprivation achieved up to 30% glutathione reduction and significantly improved the anticancer efficacy of CAP in different esophageal cancer models, suggesting a novel synergistic strategy for CAP-based cancer management. More specifically, the proportion of apoptotic cells was significantly higher in both esophageal cancer cell lines after combination therapy than the treatment of CAP alone, and the proportion of necrotic cells increased in a dose-dependent manner, suggesting that glutamine deprivation facilitates CAP treatment through another mechanism. Importantly, our work also discovered that the combined treatment could enhance oxidative damage in cells, such that MDA levels were significantly increased compared to CAP treatment or glutamine deprivation alone, possibly due to the increased intracellular ROS levels, and the excessive production of ROS (both lead to oxidative damage to critical cell membrane lipids and consequently impair cell structure and function [58].

Glutamine-deprivation caused glutathione depletion has been shown to directly regulate the formation of osmotic transition pores and the activation of cysteine aspartate protease 3 [59]. In addition, Glutamine deprivation induces apoptosis in rapidly proliferating glutamine-dependent cells such as intestinal mucosal cells and many cancer cells [60]. It has been shown that glutamine deficiency or inhibition of key regulators of glutamine metabolism, such as GLS and the transcription factor MYC, leads to prostate cancer radiosensitization [61].

Although this is an in vitro assay, further in vivo assay has been scheduled. For the next step, we will investigate changes in glutamine metabolism by up- and down-regulating glutamine transporter proteins (e.g., ASCT2, SNAT1 and SNAT2) following CAP treatment to improve our understanding about the detailed roles of glutamine in CAP-based cancer therapy. Importantly, although esophageal cancer models were used in this study, given the comparable roles of ROS and glutamine in different types of cells, the benefits of this combinatorial treatment hold potential to be expanded to the treatment of other types of cancers.

## 4. Materials and Methods

### 4.1. Cell Culture

Esophageal cancer cell lines EC9706 and ECa109 were used in this work. EC9706 cells were donated by School of Medicine in Zhengzhou University and ECa109 cells were donated by Huang Qing’s group at Hefei Institutes of Physical Sciences, Chinese Academy of Sciences. EC9706 cells grew in the DMEM medium with 10% FBS, and ECa109 cells grew in the RPMI 1640 medium with 10% FBS. Both types of cells were incubated at 37 °C in a humidified incubator with 5% CO_2_.

### 4.2. CAP Treatment

**Figure 12:**
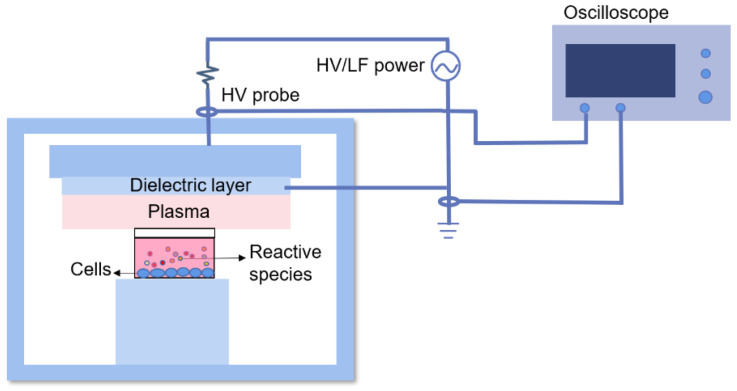
Schematic diagram of CAP device.

The CAP used in this work was created by a dielectric barrier discharge device operated at 7.50 W and 7 kHz (Figure 12). The distance between the electrode plate and the surface of the medium was kept at 1 mm. The cancer cells were exposed to the plasma for 0, 1, 3, 5 min respectively.

### 4.3. Cell Viability Assay

After treatments, cells were inoculated at 3000 cells/well in 96-well plates and incubated at 37 °C and 5% CO_2_ in a cell incubator for 24 h. At the end of the incubation, a volume of 20 µL of MTT solution (5 mg/mL) was added to each well, followed by adding each well to 200 µL using medium and incubating for an additional 4 h. The MTT treatment was then terminated by replacing the medium with 150 µL of DMSO. Cell viability was determined by measuring the absorbance at 490 nm using a microplate reader (Varioskan LUX, Thermo Scientific, Waltham, MA, USA). Each CAP treatment was performed in triplicate, while MTT was repeated three times for each sample to assess the consistency of results. The viability was calculated using the next formula OD_(sample group)_/OD_(control group)_ × 100%, as previously reported [62].

### 4.4. Quantification of NO_2_^−^

Different concentrations of NO_2_^−^ standard solutions were prepared using NaNO_2_ (analytical pure) and ddH_2_O. Firstly, adding 5 mL of cell-free medium was to a Petri dish, after CAP treatment, the media was collected, 100 µL of hydrochloric acid (1 M) and 100 µL of Sulphanilamide (10 g/L) were added and the reaction was carried out at room temperature for 5 min. After this reaction, 100 μL of N-(1-naphthy1)-ethylenediamine hydrochloride (1 g/L) was added and the reaction was carried out at room temperature for another 1 h. At the end of the reaction, 2 mL of the mixture was added to a quartz cuvette and the absorbance of the standard solution and the sample was measured at 540 nm using a UH4150 UV-Visible Spectrophotometer (Hitachi, Tokyo, Japan). A standard curve was plotted according to the absorbance of the standard solution, and the content of NO_2_^−^ in the treated medium was calculated.

### 4.5. Quantification of NO_3_^−^

The standard solution of NO_3_^−^ was prepared using NaNO_3_ (analytical pure) in DMEM medium at different concentrations from 0 mM to 2 mM. Adding 5mL medium to the Petri dish and treated with CAP, then the treated medium and standard solution were collected, 100 µL of hydrochloric acid (1 M) and 10 µL of sulfamic acid (0.8%) were added and mixed thoroughly, 2 mL of this mixed solution was added to a quartz cuvette. Then, the absorbance of the standard solution and the treatment medium was measured at 220 nm by a UH4150 UV-Visible Spectrophotometer. Finally, the absorbance of the standard solution was used to plot a standard curve and the content of NO_3_^−^ in the treated medium was calculated.

### 4.6. Quantification of H_2_O_2_

Hydrogen peroxide levels were measured using a hydrogen peroxide kit (Beyotime, Shanghai, China). The medium with or without cells were exposed to CAP and the treated cells were incubated continually for 24 h at 37 °C. After collected, the cells were fully lysed and centrifuged at 12,000× *g* for 5 min at 4 °C. The supernatant was then removed and hydrogen peroxide detection reagent was added (the treated medium without cells was analyzed directly with the reagent), and the absorbance values at 560 nm were measured immediately after the reaction using a microplate reader (Varioskan LUX, Thermo Scientific, Waltham, MA, USA) with triplicates per group.

### 4.7. Quantification of Glutathione

The cells were collected and treated with protein removal reagents. After a brief vertexing, the samples were subjected to three rapid freeze-thaw cycles using liquid nitrogen and 37 °C water. The samples were then kept at 4 °C for 5 min followed by centrifuging at 8000× *g* for 10 min at 4 °C. Then the supernatant was collected and the glutathione was analyzed using a hydrogen peroxide kit (Beyotime, Shanghai, China) according to the manufacturer’s instructions.

### 4.8. Quantification of Intracellular ROS

The cells of different groups were collected, followed by washing three times, and DCFH-DA addition at a final concentration of 10 μM. Then, the mixture was incubated for 30 min at 37 °C, and mixed upside down every 3–5 min. The fluorescence intensity was measured using a NovoCyte3 130 flow cytometer (Agilent, PAMF, Palo Alto, CA, USA) at 488 nm excitation and 525 nm emission wavelengths. Data were analyzed using FlowJo 10.6.2.

### 4.9. Quantification of MDA

The MDA was analyzed using the Malondialdehyde (MDA) Content Assay Kit (Solarbio, Beijing, China) according to the manufacturer’s instructions. At the end of the reaction, the absorbance of each sample was measured at 450 nm, 532 nm and 600 nm using a UH4150 UV-Visible Spectrophotometer (Hitachi, Tokyo, Japan). The amount of MDA was calculated according to the manufacturer’ s instructions.

### 4.10. Apoptosis Assay

The apoptosis was analyzed with Annexin V-FITC/PI Apoptosis Kit (Boster, Wuhan, China). Firstly, the cells were washed twice with pre-cooled PBS at 4 °C, followed by centrifugation (2000× *g* × 5 min). After removing the supernatant, 500 μL of binding buffer was added to resuspend cells through gentle pipetting. Afterwards, the sample was added with 5 μL Annexin V-FITC and 5 μL PI, followed by immediate mixing and incubation in the dark at room temperature for 5–15 min. Apoptosis was measured by NovoCyte3 130 flow cytometer (Agilent, PAMF, Palo Alto, CA, USA) in the FITC channel (excitation wavelength 480 nm) and the PE channel (excitation wavelength 540 nm), and the data were analyzed with FlowJo 10.6.2 software.

### 4.11. Statistical Analysis

All data were expressed as mean ± standard deviation (SD). Statistical analysis was performed using the statistical package GraphPad Prism 8. Differences between groups were determined using one-way analysis of variance (ANOVA) and compared using Duncan’s test with a confidence level of *p* < 0.05 and *p* < 0.01.

## 5. Conclusions

In this work, we found that long-lasting RONS (especially H_2_O_2_) originated from CAP stimulation increased intracellular oxidation levels and inhibited the proliferation of esophageal cancer, accompanied by the induction of apoptosis and necrosis. When a combined CAP stimulation and glutamine deprivation was performed, we further found that compared with CAP alone, the combined treatment caused reduced glutathione levels, increased intracellular ROS, and enhanced oxidative damage (e.g., increased MDA content) in the tested esophageal cancer models. Therefore, this combinatorial therapy provides a novel and effective strategy for the treatment of esophageal cancer, and even other types of cancers, given the important role of glutamine/glutathione in cancer cell metabolism.

## Data Availability

Not applicable.

## References

[1] Liu T., Fang P., Han C., Ma Z., Xu W., Xia W., Hu J., Xu Y., Xu L., Yin R. (2020). Four transcription profile-based models identify novel prognostic signatures in oesophageal cancer. J. Cell. Mol. Med..

[2] Qiao G., Zhuang W., Dong B., Li C., Xu J., Wang G., Xie L., Zhou Z., Tian D., Chen G. (2021). Discovery and validation of methylation signatures in circulating cell-free DNA for early detection of esophageal cancer: A case-control study. BMC Med..

[3] Liu X.-S., Yuan L.-L., Gao Y., Zhou L.-M., Yang J.-W., Pei Z.-J. (2020). Overexpression of METTL3 associated with the metabolic status on F-18-FDG PET/CT in patients with Esophageal Carcinoma. J. Cancer.

[4] Kadono T., Yamamoto S., Kato K. (2022). Current perspectives of the Japanese Esophageal Oncology Group on the development of immunotherapy for esophageal cancer. Jpn. J. Clin. Oncol..

[5] Lagergren J., Smyth E., Cunningham D., Lagergren P. (2017). Oesophageal cancer. Lancet.

[6] Tavares-da-Silva E., Pereira E., Pires A.S., Neves A.R., Braz-Guilherme C., Marques I.A., Abrantes A.M., Goncalves A.C., Caramelo F., Silva-Teixeira R. (2021). Cold Atmospheric Plasma, a Novel Approach against Bladder Cancer, with Higher Sensitivity for the High-Grade Cell Line. Biology.

[7] Lu X., Naidis G.V., Laroussi M., Reuter S., Graves D.B., Ostrikov K. (2016). Reactive species in non-equilibrium atmospheric-pressure plasmas: Generation, transport, and biological effects. Phys. Rep. Rev. Sect. Phys. Lett..

[8] Jawaid P., Rehman M.U., Zhao Q.-L., Misawa M., Ishikawa K., Hori M., Shimizu T., Saitoh J.-I., Noguchi K., Kondo T. (2020). Small size gold nanoparticles enhance apoptosis-induced by cold atmospheric plasma via depletion of intracellular GSH and modification of oxidative stress. Cell Death Discov..

[9] Yan D., Sherman J.H., Keidar M. (2017). Cold atmospheric plasma, a novel promising anti-cancer treatment modality. Oncotarget.

[10] Ratovitski E.A., Cheng X., Yan D., Sherman J.H., Canady J., Trink B., Keidar M. (2014). Anti-Cancer Therapies of 21st Century: Novel Approach to Treat Human Cancers Using Cold Atmospheric Plasma. Plasma Process. Polym..

[11] Biscop E., Lin A., Boxem W.V., Loenhout J.V., Backer J., Deben C., Dewilde S., Smits E., Bogaerts A.A. (2019). Influence of Cell Type and Culture Medium on Determining Cancer Selectivity of Cold Atmospheric Plasma Treatment. Cancers.

[12] Kim S.J., Chung T.H. (2016). Cold atmospheric plasma jet-generated RONS and their selective effects on normal and carcinoma cells. Sci. Rep..

[13] Yan D., Xu W., Yao X., Lin L., Sherman J.H., Keidar M. (2018). The Cell Activation Phenomena in the Cold Atmospheric Plasma Cancer Treatment. Sci. Rep..

[14] Moniruzzaman R., Rehman M.U., Zhao Q.L., Jawaid P., Takeda K., Ishikawa K., Hori M., Tomihara K., Noguchi K., Kondo T. (2017). Cold atmospheric helium plasma causes synergistic enhancement in cell death with hyperthermia and an additive enhancement with radiation. Sci. Rep..

[15] Li Y., Kang M.H., Uhm H.S., Lee G.J., Choi E.H., Han I. (2017). Effects of atmospheric-pressure non-thermal bio-compatible plasma and plasma activated nitric oxide water on cervical cancer cells. Sci. Rep..

[16] Cui H., Jiang M., Zhou W., Gao M., He R., Huang Y., Chu P.K., Yu X.F. (2021). Carrier-Free Cellular Transport of CRISPR/Cas9 Ribonucleoprotein for Genome Editing by Cold Atmospheric Plasma. Biology.

[17] Aggelopoulos C.A., Christodoulou A.M., Tachliabouri M., Meropoulis S., Christopoulou M.E., Karalis T.T., Chatzopoulos A., Skandalis S.S. (2021). Cold Atmospheric Plasma Attenuates Breast Cancer Cell Growth Through Regulation of Cell Microenvironment Effectors. Front. Oncol..

[18] Privat-Maldonado A., Verloy R., Cardenas Delahoz E., Lin A., Vanlanduit S., Smits E., Bogaerts A. (2022). Cold Atmospheric Plasma Does Not Affect Stellate Cells Phenotype in Pancreatic Cancer Tissue in Ovo. Int. J. Mol. Sci..

[19] Yusupov M., Yan D., Cordeiro R.M., Bogaerts A. (2018). Atomic scale simulation of H_2_O_2_ permeation through aquaporin: Toward the understanding of plasma cancer treatment. J. Phys. D-Appl. Phys..

[20] Yan D., Xiao H., Zhu W., Nourmohammadi N., Zhang L.G., Bian K., Keidar M. (2017). The role of aquaporins in the anti-glioblastoma capacity of the cold plasma-stimulated medium. J. Phys. D-Appl. Phys..

[21] Verkman A.S., Hara-Chikuma M., Papadopoulos M.C. (2008). Aquaporins—New players in cancer biology. J. Mol. Med. Jmm..

[22] Tong L., Chuang C.C., Wu S., Zuo L. (2015). Reactive oxygen species in redox cancer therapy. Cancer Lett..

[23] Han Y.H., Kim S.H., Kim S.Z., Park W.H. (2009). Carbonyl cyanide p-(trifluoromethoxy) phenylhydrazone (FCCP) as an O2(*−) generator induces apoptosis via the depletion of intracellular GSH contents in Calu-6 cells. Lung Cancer.

[24] Ju E., Dong K., Chen Z., Liu Z., Liu C., Huang Y., Wang Z., Pu F., Ren J., Qu X. (2016). Copper(II)-Graphitic Carbon Nitride Triggered Synergy: Improved ROS Generation and Reduced Glutathione Levels for Enhanced Photodynamic Therapy. Angew. Chem. Int. Ed..

[25] Bauer G., Sersenová D., Graves D.B., Machala Z. (2019). Dynamics of Singlet Oxygen-Triggered, RONS-Based Apoptosis Induction after Treatment of Tumor Cells with Cold Atmospheric Plasma or Plasma-Activated Medium. Sci. Rep..

[26] Park W.H. (2018). MAPK inhibitors, particularly the JNK inhibitor, increase cell death effects in H2O2-treated lung cancer cells via increased superoxide anion and glutathione depletion. Oncol. Rep..

[27] Boysen G., Jamshidi-Parsian A., Davis M.A., Siegel E.R., Simecka C.M., Kore R.A., Dings R.P.M., Griffin R.J. (2019). Glutaminase inhibitor CB-839 increases radiation sensitivity of lung tumor cells and human lung tumor xenografts in mice. Int. J. Radiat. Biol..

[28] Liu T., Sun L., Zhang Y., Wang Y., Zheng J. (2022). Imbalanced GSH/ROS and sequential cell death. J. Biochem. Mol. Toxicol..

[29] Saito K., Asai T., Fujiwara K., Sahara J., Koguchi H., Fukuda N., Suzuki-Karasaki M., Soma M., Suzuki-Karasaki Y. (2016). Tumor-selective mitochondrial network collapse induced by atmospheric gas plasma-activated medium. Oncotarget.

[30] Bandebuche S., Melinkeri R.R. (2011). Oxidative Stress and Antioxidant Status in Patients of Ovarian Cancer. Biomed. Res. India.

[31] Huang R., Chen H., Liang J., Li Y., Yang J., Luo C., Tang Y., Ding Y., Liu X., Yuan Q. (2021). Dual Role of Reactive Oxygen Species and their Application in Cancer Therapy. J. Cancer.

[32] Wang M., Holmes B., Cheng X., Zhu W., Keidar M., Zhang L.G. (2013). Cold atmospheric plasma for selectively ablating metastatic breast cancer cells. PLoS ONE.

[33] Kvam E., Davis B., Mondello F., Garner A.L. (2012). Nonthermal Atmospheric Plasma Rapidly Disinfects Multidrug-Resistant Microbes by Inducing Cell Surface Damage. Antimicrob. Agents Chemother..

[34] Ji C., Li H., Zhang L., Wang P., Lv Y., Sun Z., Tan J., Yuan Q., Tan W. (2022). Ferrocene-Containing Nucleic Acid-Based Energy-Storage Nanoagent for Continuously Photo-Induced Oxidative Stress Amplification. Angew. Chem. Int. Ed. Engl..

[35] Chen Q.-W., Liu X.-H., Fan J.-X., Peng S.-Y., Wang J.-W., Wang X.-N., Zhang C., Liu C.-J., Zhang X.-Z. (2020). Self-Mineralized Photothermal Bacteria Hybridizing with Mitochondria-T argeted Metal–Organic Frameworks for Augmenting Photothermal T umor Therapy. Adv. Funct. Mater..

[36] Kalghatgi S., Kelly C.M., Cerchar E., Torabi B., Alekseev O., Fridman A., Friedman G., Azizkhan-Clifford J. (2011). Effects of Non-Thermal Plasma on Mammalian Cells. PLoS ONE.

[37] Utsumi F., Kajiyama H., Nakamura K., Tanaka H., Hori M., Kikkawa F. (2014). Selective cytotoxicity of indirect nonequilibrium atmospheric pressure plasma against ovarian clear-cell carcinoma. Springerplus.

[38] Tornin J., Mateu-Sanz M., Rodríguez A., Labay C., Rodríguez R., Canal C. (2019). Pyruvate Plays a Main Role in the Antitumoral Selectivity of Cold Atmospheric Plasma in Osteosarcoma. Sci. Rep..

[39] Chauvin J., Judée F., Yousfi M., Vicendo P., Merbahi N. (2017). Analysis of reactive oxygen and nitrogen species generated in three liquid media by low temperature helium plasma jet. Sci. Rep..

[40] Xu D., Liu D., Wang B., Chen C., Chen Z., Li D., Yang Y., Chen H., Kong M.G. (2015). In Situ OH Generation from O2− and H2O2 Plays a Critical Role in Plasma-Induced Cell Death. PLoS ONE.

[41] Takashi Y., Tomita K., Kuwahara Y., Roudkenar M.H., Roushandeh A.M., Igarashi K., Nagasawa T., Nishitani Y., Sato T. (2020). Mitochondrial dysfunction promotes aquaporin expression that controls hydrogen peroxide permeability and ferroptosis. Free Radic. Biol. Med..

[42] Imai H., Matsuoka M., Kumagai T., Sakamoto T., Koumura T. (2017). Lipid Peroxidation-Dependent Cell Death Regulated by GPx4 and Ferroptosis. Curr. Top. Microbiol. Immunol..

[43] Shaw P., Kumar N., Privat-Maldonado A., Smits E., Bogaerts A. (2021). Cold Atmospheric Plasma Increases Temozolomide Sensitivity of Three-Dimensional Glioblastoma Spheroids via Oxidative Stress-Mediated DNA Damage. Cancers.

[44] Almeida N.D., Klein A.L., Hogan E.A., Terhaar S.J., Kedda J., Uppal P., Sack K., Keidar M., Sherman J.H. (2019). Cold Atmospheric Plasma as an Adjunct to Immunotherapy for Glioblastoma Multiforme. World Neurosurg..

[45] Köritzer J., Boxhammer V., Schäfer A., Shimizu T., Klämpfl T.G., Li Y.F., Welz C., Schwenk-Zieger S., Morfill G.E., Zimmermann J.L. (2013). Restoration of sensitivity in chemo-resistant glioma cells by cold atmospheric plasma. PLoS ONE.

[46] Soni V., Adhikari M., Lin L., Sherman J.H., Keidar M. (2022). Theranostic Potential of Adaptive Cold Atmospheric Plasma with Temozolomide to Checkmate Glioblastoma: An In Vitro Study. Cancers.

[47] Moniruzzaman R., Rehman M.U., Zhao Q.L., Jawaid P., Mitsuhashi Y., Imaue S., Fujiwara K., Ogawa R., Tomihara K., Saitoh J.I. (2018). Roles of intracellular and extracellular ROS formation in apoptosis induced by cold atmospheric helium plasma and X-irradiation in the presence of sulfasalazine. Free Radic. Biol. Med..

[48] Peng S.Y., Liu X.H., Chen Q.W., Yu Y.J., Liu M.D., Zhang X.Z. (2022). Harnessing in situ glutathione for effective ROS generation and tumor suppression via nanohybrid-mediated catabolism dynamic therapy. Biomaterials.

[49] Brunner T.F., Probst F.A., Troeltzsch M., Schwenk-Zieger S., Zimmermann J.L., Morfill G., Becker S., Harréus U., Welz C. (2022). Primary cold atmospheric plasma combined with low dose cisplatin as a possible adjuvant combination therapy for HNSCC cells-an in-vitro study. Head Face Med..

[50] Rajagopalan K.N., DeBerardinis R.J. (2011). Role of glutamine in cancer: Therapeutic and imaging implications. J. Nucl. Med..

[51] Weinberg F., Hamanaka R., Wheaton W.W., Weinberg S., Joseph J., Lopez M., Kalyanaraman B., Mutlu G.M., Budinger G.R., Chandel N.S. (2010). Mitochondrial metabolism and ROS generation are essential for Kras-mediated tumorigenicity. Proc. Natl. Acad. Sci. USA.

[52] Sun N., Liang Y., Chen Y., Wang L., Li D., Liang Z., Sun L., Wang Y., Niu H. (2019). Glutamine affects T24 bladder cancer cell proliferation by activating STAT3 through ROS and glutaminolysis. Int. J. Mol. Med..

[53] Schömel N., Hancock S.E., Gruber L., Olzomer E.M., Byrne F.L., Shah D., Hoehn K.L., Turner N., Grösch S., Geisslinger G. (2019). UGCG influences glutamine metabolism of breast cancer cells. Sci. Rep..

[54] Thomas A.G., O’Driscoll C.M., Bressler J., Kaufmann W.E., Rojas C.J., Slusher B.S. (2014). Small molecule glutaminase inhibitors block glutamate release from stimulated microglia. Biochem. Biophys. Res. Commun..

[55] Zubor P., Wang Y., Liskova A., Samec M., Koklesova L., Dankova Z., Dørum A., Kajo K., Dvorska D., Lucansky V. (2020). Cold Atmospheric Pressure Plasma (CAP) as a New Tool for the Management of Vulva Cancer and Vulvar Premalignant Lesions in Gynaecological Oncology. Int. J. Mol. Sci..

[56] Zhou R., Pantel A.R., Li S., Lieberman B.P., Ploessl K., Choi H., Blankemeyer E., Lee H., Kung H.F., Mach R.H. (2017). F-18 (2S,4R)4-Fluoroglutamine PET Detects Glutamine Pool Size Changes in Triple-Negative Breast Cancer in Response to Glutaminase Inhibition. Cancer Res..

[57] Susilo I., Maulida H., Alimsardjono L., Fauziah D., Pertiwi H. (2021). Apoptosis-Inducing Factor, Protein Expression, and Apoptosis Changes with Glutamine in Podocytes Cells Exposed with Cisplatin. Vet. Med. Int..

[58] Al-Gubory K.H. (2012). Mitochondria: Omega-3 in the route of mitochondrial reactive oxygen species. Int. J. Biochem. Cell Biol..

[59] Armstrong J.S., Jones D.P. (2002). Glutathione depletion enforces the mitochondrial permeability transition and causes cell death in HL60 cells that overexpress Bcl-2. FASEB J..

[60] Altman B.J., Stine Z.E., Dang C.V. (2016). From Krebs to clinic: Glutamine metabolism to cancer therapy. Nat. Rev. Cancer.

[61] Mukha A., Kahya U., Linge A., Chen O., Loeck S., Lukiyanchuk V., Richter S., Alves T.C., Peitzsch M., Telychko V. (2021). GLS-driven glutamine catabolism contributes to prostate cancer radiosensitivity by regulating the redox state, stemness and ATG5-mediated autophagy. Theranostics.

[62] Xue Z., Zhu J., Wang X., Yang C., Fu Z. (2021). Evaluation of the immunomodulatory effects of C9-13-CPs in macrophages. Acta Biochim. Biophys. Sin..

